# Di­chlorido­{2-[(*E*)-phen­yl(pyridin-2-yl-κ*N*)methyl­idene]-*N*-phenyl­hydra­zine­carboxamide-κ^2^ *N* ^2^,*O*}copper(II)

**DOI:** 10.1107/S1600536813026883

**Published:** 2013-10-09

**Authors:** N. Aiswarya, M. Sithambaresan, M. R. Prathapachandra Kurup, Seik Weng Ng

**Affiliations:** a Department of Applied Chemistry, Cochin University of Science and Technology, Kochi 682 022, India; b Department of Chemistry, Faculty of Science, Eastern University, Sri Lanka, Chenkalady, Sri Lanka; c Department of Chemistry, University of Malaya, 50603 Kuala Lumpur, Malaysia; d Chemistry Department, King Abdulaziz University, PO Box 80203 Jeddah, Saudi Arabia

## Abstract

The title compound, [CuCl_2_(C_19_H_16_N_4_O)], contains a Cu^II^ atom *N*,*N*′,*O*-chelated by a neutral *N*-phenyl­hy­dra­zine­car­box­amide ligand and additionally coordinated by two Cl atoms, resulting in a distorted square-pyramidal geometry. The ligating atoms in the basal square plane of the complex comprise the azomethine N, the pyridine N, the amide O and one of the Cl atoms, whereas the other Cl atom occupies an apical position. The apical Cl atoms in adjacent layers function as hydrogen-bond acceptors to both NH groups. Intermolecular C—H⋯Cl and C—H⋯O interactions are also observed.

## Related literature

For the biological applications of hydrazinecarboxamide and its derivatives, see: Beraldo & Gambino (2004 ▶); Kasuga *et al.* (2006 ▶); Rivadeneira *et al.* (2009 ▶); Shalini *et al.* (2009 ▶); Rodriguez-Arguelles *et al.* (2010 ▶). For the synthesis of related compounds, see: Kurup *et al.* (2011 ▶). For related structures, see: Kunnath *et al.* (2012 ▶). For the calculation of the trigonality index, see: Addison *et al.* (1984 ▶). For the graph-set notation, see: Etter *et al.* (1990 ▶).
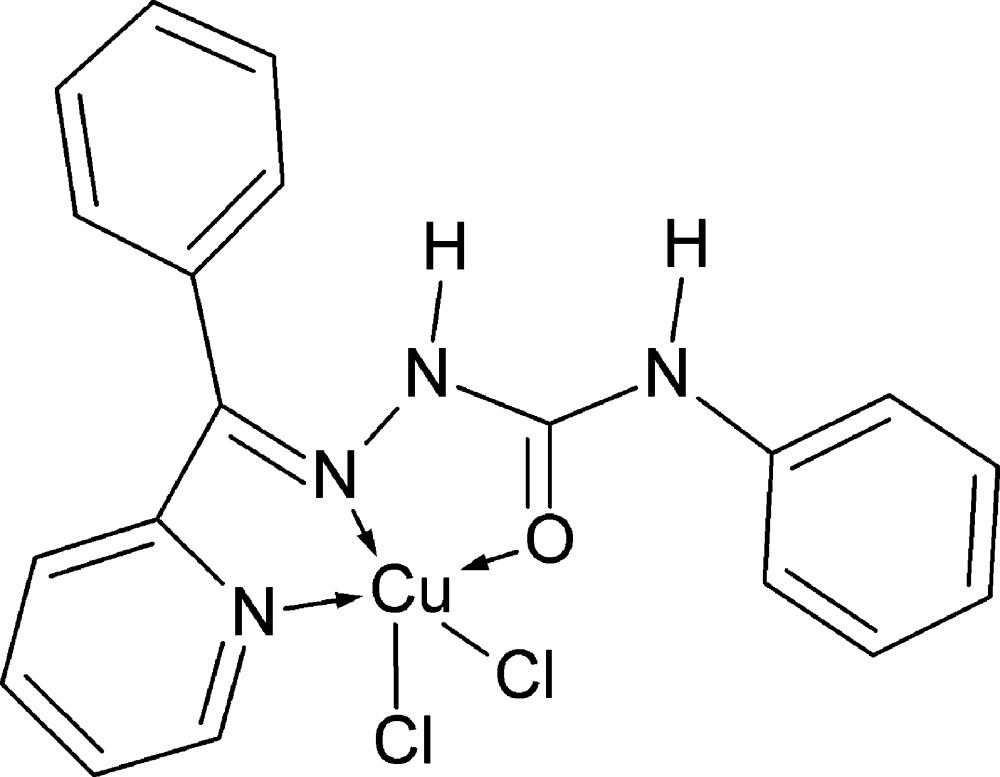


## Experimental

### 

#### Crystal data

[CuCl_2_(C_19_H_16_N_4_O)]
*M* *_r_* = 450.81
Triclinic, 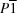

*a* = 9.4483 (5) Å
*b* = 9.8197 (3) Å
*c* = 11.5307 (4) Å
α = 104.067 (1)°
β = 103.026 (1)°
γ = 100.475 (1)°
*V* = 978.83 (7) Å^3^
*Z* = 2
Mo *K*α radiation
μ = 1.41 mm^−1^
*T* = 293 K
0.35 × 0.32 × 0.30 mm

#### Data collection

Bruker Kappa APEXII CCD diffractometer
Absorption correction: multi-scan (*SADABS*; Bruker, 2004 ▶) *T*_min_ = 0.614, *T*_max_ = 0.649
7149 measured reflections
4411 independent reflections
3550 reflections with *I* > 2σ(*I*)
*R*_int_ = 0.020

#### Refinement

*R*[*F* ^2^ > 2σ(*F* ^2^)] = 0.034
*wR*(*F* ^2^) = 0.092
*S* = 1.01
4411 reflections
252 parameters
2 restraints
H atoms treated by a mixture of independent and constrained refinement
Δρ_max_ = 0.34 e Å^−3^
Δρ_min_ = −0.40 e Å^−3^

### 

Data collection: *APEX2* (Bruker, 2004 ▶); cell refinement: *APEX2*/*SAINT* (Bruker, 2004 ▶); data reduction: *SAINT*/*XPREP* (Bruker, 2004 ▶); program(s) used to solve structure: *SHELXL97* (Sheldrick, 2008 ▶); program(s) used to refine structure: *SHELXL97* (Sheldrick, 2008 ▶); molecular graphics: *ORTEP-3* (Farrugia, 2012 ▶) and *DIAMOND* (Brandenburg, 2010 ▶); software used to prepare material for publication: *SHELXL97* and *publCIF* (Westrip, 2010 ▶).

## Supplementary Material

Crystal structure: contains datablock(s) I, global. DOI: 10.1107/S1600536813026883/fj2644sup1.cif

Structure factors: contains datablock(s) I. DOI: 10.1107/S1600536813026883/fj2644Isup2.hkl

Additional supplementary materials:  crystallographic information; 3D view; checkCIF report

## Figures and Tables

**Table 1:** Hydrogen-bond geometry (Å, °)

*D*—H⋯*A*	*D*—H	H⋯*A*	*D*⋯*A*	*D*—H⋯*A*
N3—H3′⋯Cl1^i^	0.85 (2)	2.40 (2)	3.1397 (18)	147 (2)
N4—H4′⋯Cl1^i^	0.83 (2)	2.35 (2)	3.136 (2)	159 (2)
C2—H2⋯Cl1^ii^	0.93	2.69	3.589 (4)	163
C19—H19⋯O1	0.93	2.36	2.953 (4)	121

Symmetry codes: (i) 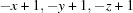; (ii) 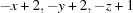.

**Fig. 1.**
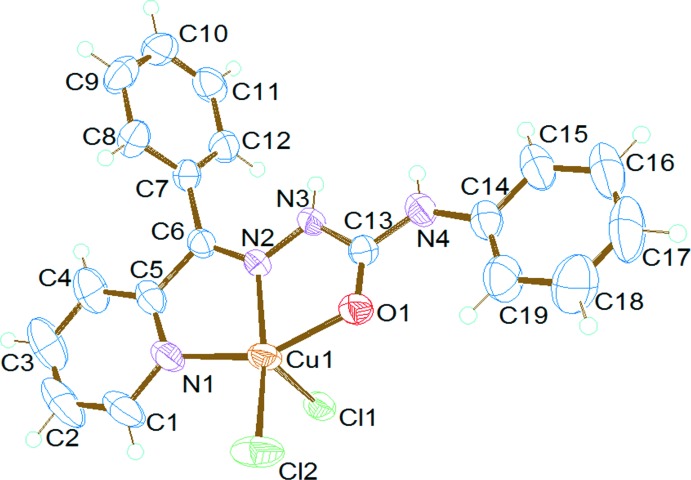
ORTEP view of the title compound, drawn with 50% probability displacement ellipsoids for the non-H atoms.

**Fig. 2.**
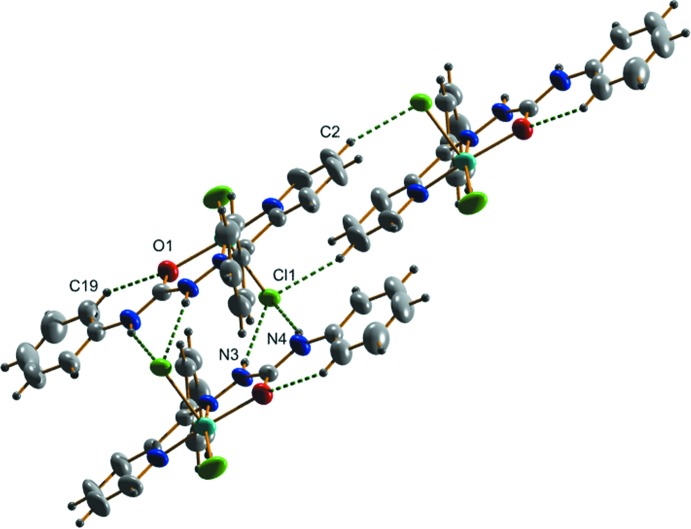
Graphical representation showing hydrogen-bonding interactions in the crystal structure of C19H16Cl2CuN4O.

**Fig. 3.**
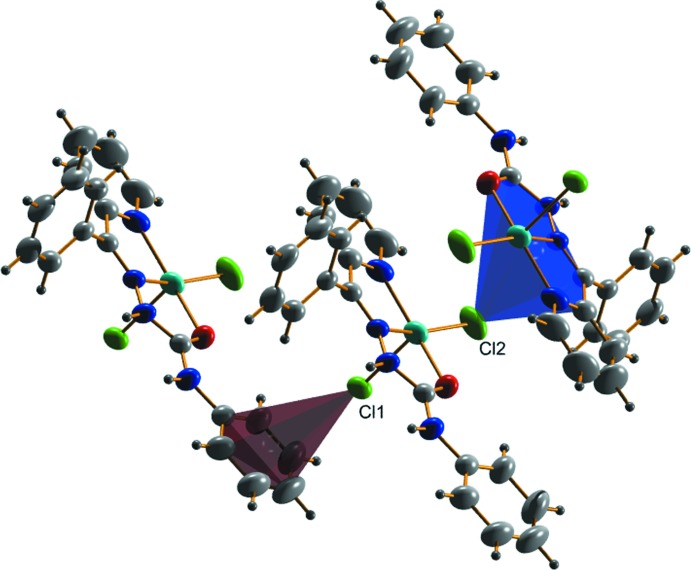
Cu—Cl···π interaction found in the title compound.

**Fig. 4.**
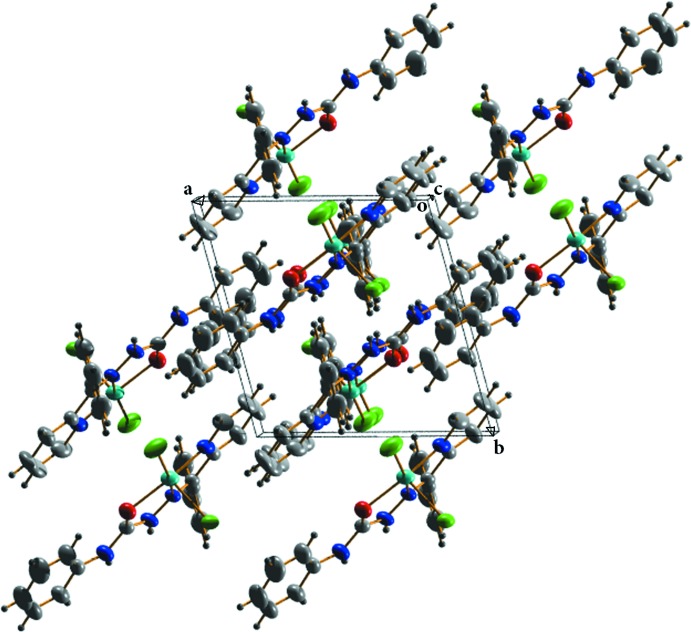
A view of the unit cell along c axis.

## supplementary crystallographic information

### 1. Comment

Semicarbazones with versatile structural features are good ligands in both the neutral and anionic forms. Among the semicarbazones with diverse pharmacological activities (Beraldo & Gambino, 2004; Kasuga *et al.*, 2006; Rivadeneira *et al.*, 2009), the aryl semicarbazones were found to be devoid of sedative hypnotic activity and exhibited anticonvulsant activity with less neurotoxicity (Shalini *et al.*, 2009). The biological activity can be attributed to their ability to form chelates with transition metal ions by bonding *via* 'O' and azomethine 'N' atoms. Also, the chlorido complex of imidazole-2-carbaldehyde semicarbazone has been found to exhibit antimicrobial activity (Rodriguez-Arguelles *et al.*, 2010).

The title compound (Fig. 1) crystallizes in the triclinic space group *P*1. The molecule adopts an *E* configuration with respect to C6═N2 bond and the tridentate ligand has its coordinating entities disposed in a *cis* fashion to each other.

The copper atom in the complex is *N*,*N*',*O* chelated by the neutral semicarbazone (Kunnath *et al.*, 2012). The C6═N2 [1.282 Å] and C13═O1 [1.229 Å] bond distances are very close to the formal C═N and C═O bond lengths [C═N; 1.28 Å and C═O; 1.21 Å] respectively confirming the azomethine bond formation and existence of semicarbazone in amido form. In addition to bond length and bond angle analysis, the trigonality index value confirms the coordination polyhedron to be a distorted square pyramidal (Addison *et al.*, 1984), with the apical chlorine atom out of the square plane by a distance of 2.450 Å. The apical chlorine atoms of the adjacent complex units function as hydrogen bond acceptors, generating a centrosymmetric dimer through a cyclic *R*_2_^1^(6) association (Etter *et al.*, 1990). In addition to that a non-classical intermolecular C–H···Cl and an intramolecular C–H···O hydrogen bonds are also present in the molecular system (Fig. 2, Table 1).

Three types of Cu—Cl···*Cg* interactions are present in the complex (Fig. 3) with *X*···*Cg* distances of 3.9266 (16), 3.5545 (11) and 3.1361 (13) Å,respectively. C—H···π and N—H···π interactions are altogether absent in the molecule. Since the *Cg*–*Cg* distances are greater than 4 Å, the short ring interactions are not significant. Fig. 4 shows the packing diagram of the title compound along *c* axis.

### 2. Experimental

The title compound was prepared by adapting a reported procedure (Kurup *et al.*, 2011). To the semicarbazone ligand synthesized by refluxing a mixture of hot methanolic solutions (25 ml) of 4-phenylsemicarbazide (0.151 g, 1 mmol) and 2-benzoylpyridine (0.183 g, 1 mmol), hot filtered methanolic solution (25 ml) of CuCl_2_·2H_2_O (0.170 g, 1 mmol) was added and refluxed for 2 h. The resulting green solution was cooled to room temperature. Green block shaped crystals were collected, washed with few drops of methanol and dried over P_4_O_10_*in vacuo*. Single crystals suitable for X-ray analysis were obtained after slow evaporation of solution in air for few days. The compound was obtained in 75% yield (0.3375 g).

### 3. Refinement

All H atoms on C were placed in calculated positions, guided by difference maps, with C—H bond distances of 0.93 Å. H atoms were assigned *U*_iso_(H) values of 1.2Ueq(carrier). Omitted owing to bad disagreement was reflection (0 0 1). H atoms of N3—H3' and N4—H4' bonds were located from difference maps and the bond distances are restrained to 0.88±0.02 Å.

### Crystal data

**Table:**

[CuCl_2_(C_19_H_16_N_4_O)]	*Z* = 2
*M**_r_* = 450.81	*F*(000) = 458
Triclinic, *P*1	*D*_x_ = 1.530 Mg m^−^^3^
Hall symbol: -P 1	Mo *K*α radiation, λ = 0.71073 Å
*a* = 9.4483 (5) Å	Cell parameters from 3151 reflections
*b* = 9.8197 (3) Å	θ = 2.5–27.7°
*c* = 11.5307 (4) Å	µ = 1.41 mm^−^^1^
α = 104.067 (1)°	*T* = 293 K
β = 103.026 (1)°	Block, green
γ = 100.475 (1)°	0.35 × 0.32 × 0.30 mm
*V* = 978.83 (7) Å^3^	

### Data collection

**Table:**

Bruker Kappa APEXII CCD diffractometer	4411 independent reflections
Radiation source: fine-focus sealed tube	3550 reflections with *I* > 2σ(*I*)
Graphite monochromator	*R*_int_ = 0.020
Detector resolution: 8.33 pixels mm^-1^	θ_max_ = 27.5°, θ_min_ = 3.3°
ω and φ scan	*h* = −9→12
Absorption correction: multi-scan (*SADABS*; Bruker, 2004)	*k* = −12→12
*T*_min_ = 0.614, *T*_max_ = 0.649	*l* = −14→14
7149 measured reflections	

### Refinement

**Table:**

Refinement on *F*^2^	Primary atom site location: structure-invariant direct methods
Least-squares matrix: full	Secondary atom site location: difference Fourier map
*R*[*F*^2^ > 2σ(*F*^2^)] = 0.034	Hydrogen site location: inferred from neighbouring sites
*wR*(*F*^2^) = 0.092	H atoms treated by a mixture of independent and constrained refinement
*S* = 1.01	*w* = 1/[σ^2^(*F*_o_^2^) + (0.0429*P*)^2^ + 0.3399*P*] where *P* = (*F*_o_^2^ + 2*F*_c_^2^)/3
4411 reflections	(Δ/σ)_max_ = 0.001
252 parameters	Δρ_max_ = 0.34 e Å^−^^3^
2 restraints	Δρ_min_ = −0.40 e Å^−^^3^

### Special details

**Table:**

Geometry. All e.s.d.'s (except the e.s.d. in the dihedral angle between two l.s. planes) are estimated using the full covariance matrix. The cell e.s.d.'s are taken into account individually in the estimation of e.s.d.'s in distances, angles and torsion angles; correlations between e.s.d.'s in cell parameters are only used when they are defined by crystal symmetry. An approximate (isotropic) treatment of cell e.s.d.'s is used for estimating e.s.d.'s involving l.s. planes.
Refinement. Refinement of *F*^2^ against ALL reflections. The weighted *R*-factor *wR* and goodness of fit *S* are based on *F*^2^, conventional *R*-factors *R* are based on *F*, with *F* set to zero for negative *F*^2^. The threshold expression of *F*^2^ > σ(*F*^2^) is used only for calculating *R*-factors(gt) *etc*. and is not relevant to the choice of reflections for refinement. *R*-factors based on *F*^2^ are statistically about twice as large as those based on *F*, and *R*- factors based on ALL data will be even larger.

### Fractional atomic coordinates and isotropic or equivalent isotropic displacement parameters (Å^2^)

**Table:**

	*x*	*y*	*z*	*U*_iso_*/*U*_eq_	
Cu1	0.56784 (3)	0.81865 (3)	0.45210 (3)	0.04217 (10)	
Cl1	0.68970 (7)	0.63456 (5)	0.34892 (5)	0.04308 (14)	
Cl2	0.54712 (10)	0.95006 (8)	0.32265 (7)	0.0657 (2)	
O1	0.35179 (18)	0.68219 (17)	0.38879 (14)	0.0435 (4)	
N1	0.7528 (2)	0.9491 (2)	0.5849 (2)	0.0474 (5)	
N2	0.5556 (2)	0.74344 (17)	0.59382 (16)	0.0343 (4)	
N3	0.4287 (2)	0.64128 (19)	0.57491 (17)	0.0385 (4)	
H3'	0.418 (3)	0.593 (2)	0.625 (2)	0.048 (7)*	
N4	0.2064 (2)	0.5040 (2)	0.43869 (19)	0.0459 (5)	
C1	0.8558 (4)	1.0534 (3)	0.5712 (3)	0.0691 (9)	
H1	0.8419	1.0733	0.4952	0.083*	
C2	0.9811 (4)	1.1309 (4)	0.6673 (5)	0.0930 (12)	
H2	1.0514	1.2026	0.6563	0.112*	
C3	1.0019 (4)	1.1029 (4)	0.7775 (4)	0.0938 (12)	
H3	1.0865	1.1555	0.8431	0.113*	
C4	0.8967 (3)	0.9953 (3)	0.7934 (3)	0.0712 (8)	
H4	0.9098	0.9743	0.8690	0.085*	
C5	0.7732 (3)	0.9209 (2)	0.6946 (2)	0.0450 (5)	
C6	0.6523 (2)	0.8054 (2)	0.7004 (2)	0.0358 (4)	
C7	0.6428 (2)	0.7762 (2)	0.81791 (19)	0.0381 (5)	
C8	0.6340 (3)	0.8871 (3)	0.9153 (2)	0.0500 (6)	
H8	0.6375	0.9793	0.9066	0.060*	
C9	0.6203 (3)	0.8607 (3)	1.0242 (2)	0.0596 (7)	
H9	0.6128	0.9348	1.0884	0.071*	
C10	0.6176 (3)	0.7256 (3)	1.0393 (2)	0.0587 (7)	
H10	0.6090	0.7088	1.1137	0.070*	
C11	0.6275 (3)	0.6155 (3)	0.9440 (2)	0.0564 (7)	
H11	0.6263	0.5244	0.9544	0.068*	
C12	0.6394 (3)	0.6395 (3)	0.8329 (2)	0.0458 (5)	
H12	0.6450	0.5644	0.7684	0.055*	
C13	0.3276 (2)	0.6125 (2)	0.46062 (19)	0.0364 (5)	
C14	0.0774 (3)	0.4486 (3)	0.3355 (2)	0.0467 (5)	
C15	−0.0277 (3)	0.3329 (3)	0.3403 (3)	0.0649 (8)	
H15	−0.0097	0.2966	0.4081	0.078*	
C16	−0.1589 (4)	0.2715 (4)	0.2447 (4)	0.0832 (10)	
H16	−0.2297	0.1949	0.2488	0.100*	
C17	−0.1846 (4)	0.3227 (5)	0.1446 (4)	0.0988 (13)	
H17	−0.2719	0.2796	0.0793	0.119*	
C18	−0.0819 (4)	0.4383 (5)	0.1398 (4)	0.0940 (12)	
H18	−0.1011	0.4743	0.0719	0.113*	
C19	0.0509 (3)	0.5020 (3)	0.2357 (3)	0.0664 (8)	
H19	0.1206	0.5798	0.2319	0.080*	
H4'	0.210 (3)	0.465 (3)	0.496 (2)	0.050 (8)*	

### Atomic displacement parameters (Å^2^)

**Table:**

	*U*^11^	*U*^22^	*U*^33^	*U*^12^	*U*^13^	*U*^23^
Cu1	0.05420 (19)	0.03721 (16)	0.04289 (17)	0.00880 (12)	0.02232 (13)	0.01988 (12)
Cl1	0.0519 (3)	0.0338 (3)	0.0490 (3)	0.0081 (2)	0.0230 (3)	0.0161 (2)
Cl2	0.0992 (6)	0.0637 (4)	0.0735 (4)	0.0432 (4)	0.0528 (4)	0.0476 (4)
O1	0.0447 (9)	0.0477 (9)	0.0384 (8)	0.0059 (7)	0.0103 (7)	0.0190 (7)
N1	0.0500 (12)	0.0343 (9)	0.0629 (13)	0.0037 (9)	0.0314 (10)	0.0142 (9)
N2	0.0371 (10)	0.0284 (8)	0.0358 (9)	0.0010 (7)	0.0125 (8)	0.0099 (7)
N3	0.0438 (11)	0.0336 (9)	0.0337 (9)	−0.0034 (8)	0.0093 (8)	0.0135 (7)
N4	0.0431 (11)	0.0439 (11)	0.0445 (11)	−0.0019 (9)	0.0069 (9)	0.0167 (9)
C1	0.069 (2)	0.0500 (15)	0.102 (2)	0.0047 (14)	0.0523 (19)	0.0291 (15)
C2	0.057 (2)	0.063 (2)	0.156 (4)	−0.0107 (16)	0.044 (2)	0.033 (2)
C3	0.0433 (18)	0.080 (2)	0.129 (3)	−0.0197 (16)	0.004 (2)	0.024 (2)
C4	0.0431 (16)	0.0650 (18)	0.087 (2)	−0.0045 (13)	0.0021 (15)	0.0175 (16)
C5	0.0352 (12)	0.0374 (11)	0.0601 (15)	0.0044 (9)	0.0168 (11)	0.0107 (10)
C6	0.0362 (11)	0.0304 (10)	0.0405 (11)	0.0067 (8)	0.0133 (9)	0.0089 (8)
C7	0.0337 (11)	0.0398 (11)	0.0348 (11)	0.0055 (9)	0.0043 (9)	0.0081 (9)
C8	0.0583 (16)	0.0470 (13)	0.0391 (12)	0.0146 (12)	0.0068 (11)	0.0080 (10)
C9	0.0671 (19)	0.0687 (18)	0.0319 (12)	0.0138 (14)	0.0067 (12)	0.0041 (11)
C10	0.0535 (16)	0.081 (2)	0.0347 (12)	0.0047 (14)	0.0041 (11)	0.0220 (13)
C11	0.0546 (16)	0.0560 (15)	0.0559 (15)	0.0024 (12)	0.0057 (12)	0.0291 (13)
C12	0.0495 (14)	0.0413 (12)	0.0434 (12)	0.0069 (10)	0.0105 (11)	0.0124 (10)
C13	0.0398 (12)	0.0319 (10)	0.0367 (11)	0.0070 (9)	0.0122 (9)	0.0089 (8)
C14	0.0369 (13)	0.0430 (12)	0.0529 (14)	0.0084 (10)	0.0097 (11)	0.0049 (10)
C15	0.0482 (16)	0.0527 (15)	0.080 (2)	−0.0010 (13)	0.0132 (14)	0.0086 (14)
C16	0.0487 (18)	0.070 (2)	0.099 (3)	−0.0065 (15)	0.0032 (18)	−0.0016 (19)
C17	0.051 (2)	0.099 (3)	0.099 (3)	0.0014 (19)	−0.0168 (19)	−0.008 (2)
C18	0.072 (2)	0.114 (3)	0.077 (2)	0.022 (2)	−0.0126 (19)	0.026 (2)
C19	0.0487 (16)	0.0738 (19)	0.0654 (18)	0.0083 (14)	−0.0001 (13)	0.0205 (15)

### Geometric parameters (Å, º)

**Table:**

Cu1—N2	1.9672 (16)	C6—C7	1.470 (3)
Cu1—N1	2.016 (2)	C7—C12	1.390 (3)
Cu1—O1	2.0865 (16)	C7—C8	1.391 (3)
Cu1—Cl2	2.1973 (6)	C8—C9	1.370 (4)
Cu1—Cl1	2.5175 (6)	C8—H8	0.9300
O1—C13	1.229 (3)	C9—C10	1.376 (4)
N1—C5	1.339 (3)	C9—H9	0.9300
N1—C1	1.344 (3)	C10—C11	1.375 (4)
N2—C6	1.282 (3)	C10—H10	0.9300
N2—N3	1.352 (2)	C11—C12	1.383 (3)
N3—C13	1.372 (3)	C11—H11	0.9300
N3—H3'	0.846 (16)	C12—H12	0.9300
N4—C13	1.343 (3)	C14—C19	1.369 (4)
N4—C14	1.410 (3)	C14—C15	1.388 (4)
N4—H4'	0.833 (16)	C15—C16	1.380 (4)
C1—C2	1.371 (5)	C15—H15	0.9300
C1—H1	0.9300	C16—C17	1.358 (5)
C2—C3	1.344 (6)	C16—H16	0.9300
C2—H2	0.9300	C17—C18	1.374 (6)
C3—C4	1.389 (4)	C17—H17	0.9300
C3—H3	0.9300	C18—C19	1.393 (4)
C4—C5	1.371 (4)	C18—H18	0.9300
C4—H4	0.9300	C19—H19	0.9300
C5—C6	1.483 (3)		
			
N2—Cu1—N1	78.70 (7)	C7—C6—C5	122.45 (19)
N2—Cu1—O1	77.84 (6)	C12—C7—C8	119.4 (2)
N1—Cu1—O1	154.03 (7)	C12—C7—C6	121.4 (2)
N2—Cu1—Cl2	162.21 (6)	C8—C7—C6	119.2 (2)
N1—Cu1—Cl2	99.08 (6)	C9—C8—C7	120.0 (2)
O1—Cu1—Cl2	99.86 (5)	C9—C8—H8	120.0
N2—Cu1—Cl1	96.69 (5)	C7—C8—H8	120.0
N1—Cu1—Cl1	97.98 (6)	C8—C9—C10	120.7 (2)
O1—Cu1—Cl1	95.64 (5)	C8—C9—H9	119.6
Cl2—Cu1—Cl1	101.09 (2)	C10—C9—H9	119.6
C13—O1—Cu1	112.00 (14)	C11—C10—C9	119.7 (2)
C5—N1—C1	119.2 (3)	C11—C10—H10	120.1
C5—N1—Cu1	114.21 (15)	C9—C10—H10	120.1
C1—N1—Cu1	126.5 (2)	C10—C11—C12	120.5 (2)
C6—N2—N3	123.84 (17)	C10—C11—H11	119.8
C6—N2—Cu1	120.05 (14)	C12—C11—H11	119.8
N3—N2—Cu1	115.29 (13)	C11—C12—C7	119.7 (2)
N2—N3—C13	113.53 (17)	C11—C12—H12	120.1
N2—N3—H3'	122.5 (18)	C7—C12—H12	120.1
C13—N3—H3'	123.5 (18)	O1—C13—N4	125.9 (2)
C13—N4—C14	129.7 (2)	O1—C13—N3	120.75 (19)
C13—N4—H4'	113.5 (19)	N4—C13—N3	113.39 (19)
C14—N4—H4'	116.8 (19)	C19—C14—C15	119.9 (3)
N1—C1—C2	121.2 (3)	C19—C14—N4	124.6 (2)
N1—C1—H1	119.4	C15—C14—N4	115.5 (2)
C2—C1—H1	119.4	C16—C15—C14	120.2 (3)
C3—C2—C1	119.8 (3)	C16—C15—H15	119.9
C3—C2—H2	120.1	C14—C15—H15	119.9
C1—C2—H2	120.1	C17—C16—C15	120.1 (3)
C2—C3—C4	119.9 (3)	C17—C16—H16	120.0
C2—C3—H3	120.1	C15—C16—H16	120.0
C4—C3—H3	120.1	C16—C17—C18	120.1 (3)
C5—C4—C3	118.2 (3)	C16—C17—H17	120.0
C5—C4—H4	120.9	C18—C17—H17	120.0
C3—C4—H4	120.9	C17—C18—C19	120.6 (4)
N1—C5—C4	121.8 (2)	C17—C18—H18	119.7
N1—C5—C6	114.9 (2)	C19—C18—H18	119.7
C4—C5—C6	123.3 (2)	C14—C19—C18	119.1 (3)
N2—C6—C7	125.61 (19)	C14—C19—H19	120.4
N2—C6—C5	111.80 (19)	C18—C19—H19	120.4
			
N2—Cu1—O1—C13	−6.89 (15)	N3—N2—C6—C5	−175.36 (19)
N1—Cu1—O1—C13	−32.7 (3)	Cu1—N2—C6—C5	−6.2 (2)
Cl2—Cu1—O1—C13	−168.91 (14)	N1—C5—C6—N2	6.1 (3)
Cl1—Cu1—O1—C13	88.77 (15)	C4—C5—C6—N2	−174.4 (2)
N2—Cu1—N1—C5	0.20 (16)	N1—C5—C6—C7	−169.7 (2)
O1—Cu1—N1—C5	25.9 (3)	C4—C5—C6—C7	9.7 (4)
Cl2—Cu1—N1—C5	162.26 (16)	N2—C6—C7—C12	61.0 (3)
Cl1—Cu1—N1—C5	−95.08 (16)	C5—C6—C7—C12	−123.7 (2)
N2—Cu1—N1—C1	177.3 (2)	N2—C6—C7—C8	−117.7 (3)
O1—Cu1—N1—C1	−157.0 (2)	C5—C6—C7—C8	57.6 (3)
Cl2—Cu1—N1—C1	−20.6 (2)	C12—C7—C8—C9	−0.8 (4)
Cl1—Cu1—N1—C1	82.0 (2)	C6—C7—C8—C9	177.9 (2)
N1—Cu1—N2—C6	3.66 (16)	C7—C8—C9—C10	1.1 (4)
O1—Cu1—N2—C6	−165.14 (18)	C8—C9—C10—C11	−0.5 (4)
Cl2—Cu1—N2—C6	−80.8 (3)	C9—C10—C11—C12	−0.4 (4)
Cl1—Cu1—N2—C6	100.51 (16)	C10—C11—C12—C7	0.7 (4)
N1—Cu1—N2—N3	173.68 (16)	C8—C7—C12—C11	−0.1 (4)
O1—Cu1—N2—N3	4.88 (14)	C6—C7—C12—C11	−178.8 (2)
Cl2—Cu1—N2—N3	89.2 (2)	Cu1—O1—C13—N4	−171.66 (19)
Cl1—Cu1—N2—N3	−89.48 (14)	Cu1—O1—C13—N3	8.0 (3)
C6—N2—N3—C13	167.1 (2)	C14—N4—C13—O1	−3.4 (4)
Cu1—N2—N3—C13	−2.5 (2)	C14—N4—C13—N3	176.9 (2)
C5—N1—C1—C2	0.1 (4)	N2—N3—C13—O1	−4.0 (3)
Cu1—N1—C1—C2	−176.9 (2)	N2—N3—C13—N4	175.67 (19)
N1—C1—C2—C3	−0.1 (6)	C13—N4—C14—C19	−1.9 (4)
C1—C2—C3—C4	0.2 (6)	C13—N4—C14—C15	178.9 (3)
C2—C3—C4—C5	−0.3 (6)	C19—C14—C15—C16	0.0 (5)
C1—N1—C5—C4	−0.2 (4)	N4—C14—C15—C16	179.3 (3)
Cu1—N1—C5—C4	177.2 (2)	C14—C15—C16—C17	1.0 (5)
C1—N1—C5—C6	179.3 (2)	C15—C16—C17—C18	−1.7 (6)
Cu1—N1—C5—C6	−3.4 (3)	C16—C17—C18—C19	1.4 (7)
C3—C4—C5—N1	0.3 (5)	C15—C14—C19—C18	−0.3 (5)
C3—C4—C5—C6	−179.1 (3)	N4—C14—C19—C18	−179.5 (3)
N3—N2—C6—C7	0.4 (3)	C17—C18—C19—C14	−0.4 (6)
Cu1—N2—C6—C7	169.48 (16)		

### Hydrogen-bond geometry (Å, º)

**Table:**

*D*—H···*A*	*D*—H	H···*A*	*D*···*A*	*D*—H···*A*
N3—H3′···Cl1^i^	0.85 (2)	2.40 (2)	3.1397 (18)	147 (2)
N4—H4′···Cl1^i^	0.83 (2)	2.35 (2)	3.136 (2)	159 (2)
C2—H2···Cl1^ii^	0.93	2.69	3.589 (4)	163
C19—H19···O1	0.93	2.36	2.953 (4)	121

Symmetry codes: (i) −*x*+1, −*y*+1, −*z*+1; (ii) −*x*+2, −*y*+2, −*z*+1.
